# Genetic Basis of Inflammatory Demyelinating Diseases of the Central Nervous System: Multiple Sclerosis and Neuromyelitis Optica Spectrum

**DOI:** 10.3390/genes14071319

**Published:** 2023-06-23

**Authors:** Genaro Gabriel Ortiz, Blanca M. G. Torres-Mendoza, Javier Ramírez-Jirano, Jazmin Marquez-Pedroza, José J. Hernández-Cruz, Mario A. Mireles-Ramirez, Erandis D. Torres-Sánchez

**Affiliations:** 1 Department of Philosophical and Methodological Disciplines and Service of Molecular Biology in Medicine Hospital, Civil University Health Sciences Center, University of Guadalajara, Guadalajara 44340, Jalisco, Mexico; genarogabriel@yahoo.com (G.G.O.); bltorres1@hotmail.com (B.M.G.T.-M.); 2 Department of Neurology, High Specialty Medical Unit, Western National Medical Center of the Mexican Institute of Social Security, Guadalajara 44329, Jalisco, Mexico; jojeshercruz@gmail.com; 3 Neurosciences Division, Western Biomedical Research Center, Mexican Social Security Institute (Instituto Mexicano del Seguro Social, IMSS), Guadalajara 44340, Jalisco, Mexico; ramirez_jirano@hotmail.com (J.R.-J.); jaz180688@gmail.com (J.M.-P.); 4 Coordination of Academic Activities, Western Biomedical Research Center, Mexican Social Security Institute (Instituto Mexicano del Seguro Social, IMSS), Guadalajara 44340, Jalisco, Mexico; 5 Department of Medical and Life Sciences, University Center of la Cienega, University of Guadalajara, Ocotlan 47820, Jalisco, Mexico

**Keywords:** multiple sclerosis, neuromyelitis optica spectrum disorders, genetic basis, inflammatory demyelinating disease, genes

## Abstract

Demyelinating diseases alter myelin or the coating surrounding most nerve fibers in the central and peripheral nervous systems. The grouping of human central nervous system demyelinating disorders today includes multiple sclerosis (MS) and neuromyelitis optica spectrum disorders (NMOSD) as distinct disease categories. Each disease is caused by a complex combination of genetic and environmental variables, many involving an autoimmune response. Even though these conditions are fundamentally similar, research into genetic factors, their unique clinical manifestations, and lesion pathology has helped with differential diagnosis and disease pathogenesis knowledge. This review aims to synthesize the genetic approaches that explain the differential susceptibility between these diseases, explore the overlapping clinical features, and pathological findings, discuss existing and emerging hypotheses on the etiology of demyelination, and assess recent pathogenicity studies and their implications for human demyelination. This review presents critical information from previous studies on the disease, which asks several questions to understand the gaps in research in this field.

## 1. Introduction

Demyelinating diseases are a group of pathologies that alter myelin or the coating surrounding most nerve fibers in the central and peripheral nervous systems. Each nerve fiber comprises an axon and the myelin that covers it. This lining consists of proteins, lipids, and several layers, forming a myelin sheath. The function of this sheath is to insulate the axons that it covers and allow the conduction of nerve impulses to and from the brain quickly (in fact, there are also unmyelinated fibers along which conduction is slower) [1]. When the myelin sheath is irreversibly damaged, electrical signal conduction can no longer occur, and nerves (composed of multiple bundles of nerve fibers) are damaged. Multiple sclerosis (MS), neuromyelitis optica spectrum disease (NMOSD), adrenoleukodystrophy, acute disseminated encephalomyelitis, adrenomyeloneuropathy, and Leber’s hereditary optic neuropathy are demyelinating diseases [2,3].

MS is an autoimmune disease characterized by brain, spinal cord, and optic pathways damage with significant clinical polymorphism. Globally, the number of people affected by MS is estimated at 2.5 million [1]. It is the leading cause of non-traumatic acquired motor disability in young adults in the West. It begins in approximately 70% of cases between the ages of 20 and 40 [1]. MS is more common in women than in men [1], unlike some diseases, such as hemophilia or muscle diseases [3]. Several factors have been implicated in MS pathogenesis: the role of genetics on the one hand, the influence of environmental factors on the other, such as sunlight and vitamin D, smoking or the Epstein–Barr virus (Table 1). Although the etiology is not precisely clarified, it is accepted that the immune system plays an important role in the appearance of the disease, each of its actors having a significant influence [4]. The research improves our knowledge of this disease and guides research toward new therapies, particularly for the progressive form. MS is characterized by two events: inflammatory flare-ups and disability progression. The clinical forms can be separated into two main progressive types: relapsing forms and progressive forms. The relapsing forms are the most frequent since they represent 85% of the cases, preferentially affecting women. They are characterized by the appearance of inflammatory attacks that lead to a progression of disability. After approximately ten years, the relapsing-remitting MS form (RR-MS) can progress to a secondary progressive form (SP-MS). The primary progressive form (PP-MS) is characterized by a progressive and irreversible course without a period of calm. It affects 15% of patients with MS, both men and women, and is manifested mainly by a progressive evolution of disability that includes gait disorders [4,5].

NMOSD, also called Devic’s disease, is characterized by severe optic neuritis, which can be a bilateral, transverse myelitis of longitudinal extension, affecting at least three vertebral segments, with few brain lesions. At the beginning, however, Sahraian and collaborators find brain abnormalities in 50 to 85% of cases during the course. The differential diagnosis with MS is therefore sometimes difficult. The appearance of the two clinical elements and the absence of magnetic resonance imaging (MRI) abnormalities characteristic of MS guides the diagnosis [19]. In 2004, an anti-AQP4 antibody was described as specific for neuromyelitis optica (NMO). This biomarker would be present in 50–70% of NMO and allows a precise differential diagnosis with MS [20]. The notion of an inflammation of the nervous system that mainly affects the optic nerves and the spinal cord is the basis of the concept of NMO. Already, in 1970, Thomas Clifford Allbut was surprised by the frequent association between acute myelitis and ocular involvement [21]. The German pathologist Julius Dreschfeld reported in 1982 the case of a patient who associated paraplegia and marked optic neuritis [22]. He described a softened, yellowish cervical cord lesion, extending 1 1⁄2 inches, which was associated with typical neuritis, matching to the highest degree that which Uhtoff described in chronic alcoholism while the brain was completely healthy. It was in 1894 that Eugène Devic and his student Fernand Gault first described Devic’s NMO [23,24]. Myelitis sets in gradually but in a cycle that leads to complete regression if respiratory disorders are not fatal. Anatomically, NMO lesions are necrotic and demyelinating, located in the anterior optic pathways and a spinal segment, and may extend to the entire spinal cord but not spread [25].

Demyelination is often secondary to an infectious disease, ischemic disease, metabolic disorder, hereditary disorder, or a toxin (e.g., alcohol, ethambutol). In primary demyelinating diseases, the cause is unknown, but an autoimmune mechanism is suspected, as the disease sometimes follows a viral infection or vaccination against a virus. Demyelination tends to be segmental or patchy, affecting multiple areas simultaneously or sequentially. Remyelination often occurs with repair, regeneration, and the complete recovery of neuronal function. However, extensive myelin loss is usually followed by the degeneration of the axon and often the cell body; both can be irreversible [2].

The hypothesis of a demyelinating disease should be suspected in any patient with neurological deficits that cannot be explained otherwise. Primary demyelinating diseases should be presumed in the presence of:

- Diffuse or multifocal deficit;
- Sudden or subacute onset, particularly in young adults;
- Onset within a few weeks of infection or vaccination;
- Relapsing-remitting symptoms;
- Symptoms suggestive of a specific demyelinating disease (e.g., unexplained optic neuritis or internuclear ophthalmoplegia suggestive of multiple sclerosis) [2,3].

The neurological symptoms vary according to the demyelinated nerve fibers, the disease, and its progression. They include muscle weakness, involuntary muscle contractions (spasticity), loss of motor coordination, paralysis, loss of sensation, tingling, vision problems or even blindness, hearing problems, slurred speech, incontinence, depression, nausea, headaches, and fever, among others [3].

On the treatment side, current therapies positively impact the disease’s progression, thanks to a vast therapeutic panorama, which is not only pharmacological. For this reason, the treatment decision must be made based on current advances in therapies since such a decision significantly affects the curse of the disease. We are in the context of personalized medicine, which is a comprehensive and prospective approach to prevention, diagnosis, treatment, and follow-up based on the unique individual, carried out in a multidisciplinary group, from the neurologist to the psychologist, from the physiotherapist to the nutritionist, from the nurse to several other consultants, in which students, clinicians, and researchers can find new stimuli. Please find out more about the narrative that we are writing to create a world free of multiple sclerosis and similar conditions [2,3].

Among the arguments in favor of genetic susceptibility to these diseases, we find that their distribution is not uniform throughout the world and is more according to the different ethnic groups of the same geographical region [2,26,27]. The observation of multiple cases in the same family reinforces this hypothesis. The children of a person with MS or NMOSD have a higher risk of developing this disease than the general population [26].

## 2. Epidemiology and Prevalence of MS

The geographic distribution of the prevalence of MS is not homogeneous [14,28,29]. Studies show that MS is more common in temperate regions (more than 100 to 200 cases per 100,000 people) than in tropical areas (fewer than 5 cases per 100,000 people). In the north of Scotland, 250 cases per 100,000 inhabitants are reported, and this similar risk is found in northern Europe and North America, which are the most affected areas. The areas with the lowest prevalence risk (with 6 cases per 100,000 inhabitants) are Japan, North Africa, and the Middle East. Thus, we observe that the distribution of the disease is not uniform: from a geographical point of view, we observe a north–south gradient, with the countries furthest from the equator being those with the highest risk. Industrialized countries, as well as northern European countries, are more frequently affected. Nevertheless, it is worth noting a complete lack of data for much of the African continent and several Middle Eastern countries [1,30]. However, the long-held notion of a north–south MS prevalence gradient is now in question. Only the Australian population, considered homogeneous because it descended from the English people, seems to present this north–south distribution [31]. Epidemiological studies have also shown variation in individual risk according to place of birth, ethnicity, and whether there is another case in the family [5]. The data’s lack of precision and homogeneity greatly diminishes the power and importance of these epidemiological studies. In fact, it has been mentioned that many biases can lead to the conclusion of false differences in prevalence between two countries. For example, different diagnostic criteria, more difficult access to medical specialists in certain countries or certain regions of a country, and incomplete national databases can lead to different prevalence figures [32]. All this means that, even today, it is difficult to say whether environment or genetics is the main cause of the differences in the prevalence of the disease that are observed between different ethnic groups, countries, and even different regions within the same country.

MS is a disease of young adults that preferentially affects women, generally between the ages of 25 and 35. However, rare pediatric forms of the disease exist: 3–10% of MS patients are younger than 18 years of age and 0.2–0.4% are younger than 10 years of age, with an average age of 12 years at diagnosis. The statistics for the disease’s primary progressive form differ from the relapsing form: men and women are affected equally, and the age of disease onset is around 40 years. A similar age to that of RR-MS can convert to a secondary progressive form [1,5].

## 3. Influence of Migration in MS

**Figure 1:**
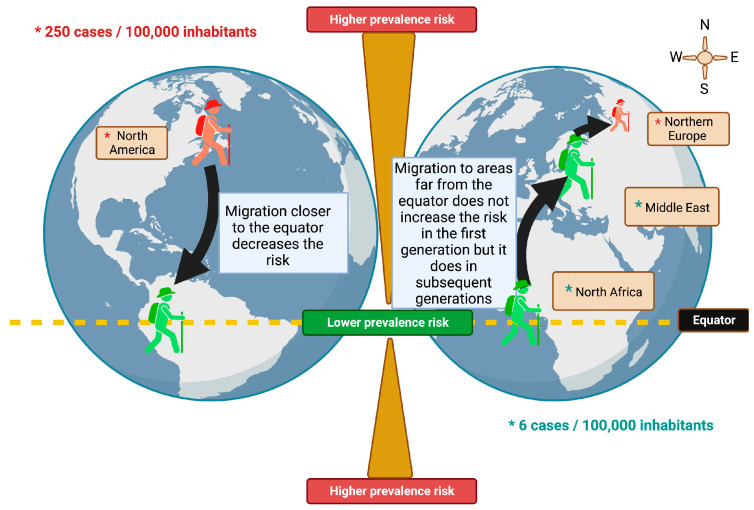
Risk in the development of MS through migration. Greater risk is observed in people in remote areas of the equator. Created with Biorender.com.

Migration studies highlight for the first time the influence of environmental factors on the probability of developing MS. Many studies have been carried out on the subject and the first conclusions showed two patterns: people who migrate from an area with a high prevalence to an area where it is relatively rare, see their risk decrease, while, on the contrary, people migrating from a low-risk area to a high-risk area seemed to maintain the low risk of their country of origin. However, this low risk was kept only for the first generation, as subsequent generations tended to acquire the risk of their host country, suggesting that the individual risk of developing MS was established within the first two decades of life (Figure 1) [33].

Population migration studies have shown that people who migrate from high-risk to low-risk areas have a lower risk of developing the disease. However, this is not otherwise verified. Immigrants who come from a country with a low risk of MS and settle in a country with a high risk of MS tend to maintain a low risk of developing the disease compared to that in their country of origin [34]. People from the United Kingdom and Ireland (UK-I), and migrants, coming from a high prevalence area (UK-I) to a lower prevalence area (Australia), who are aged less than 15, are significantly less likely to develop the disease than those older people [35]. Thus, the risk of developing MS would vary after migration and this variation seems to depend on the age of departure. Those who migrate after the age of 15 tend to retain the risk of their country of birth, while those who migrate before that age tend to acquire the risk of their host country; suggesting that childhood, and adolescence are critical periods influenced by the environment. These studies that include the migration age of people sought to determine if there was a threshold age from which the risk of developing MS was established. Although studies seem to set an age threshold of approximately 15 years, others have not shown a correlation between migration age and the risk of developing MS [14].

## 4. Factors That Increase the Risk of MS

MS is a complex disease involving many genetic and environmental factors. Therefore, it develops in predisposed subjects after interaction with exogenous factors. Among the defendants, we can mention the sun and the concentration of vitamin D, life habits, and a possible infectious cause with the Epstein–Barr virus, (EBV), the agent responsible for infectious mononucleosis [6].

Other environmental factors are suspected to play a role in triggering the disease, such as tobacco, fats (diet), or certain chemical compounds (Table 1) [12,14], but no study to date has been able to implicate them in a formal way. Some studies seem to highlight a role for the intestinal microbiota. A change in the gut microbiota has been shown to alter the clinical features of EAE [36].

**Figure 2:**
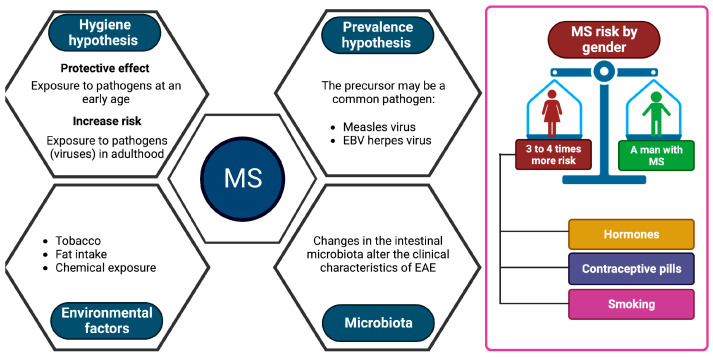
Factors involved in MS development. Key factors that increase the risk of multiple sclerosis include virus-associated pathologies, exposure to endogenous substances, such as those indicated, and the alteration of the microbiota. Created with Biorender.com.

MS is more common in women, with 3 to 4 women affected for every man affected [5,37]. Some studies show that this increased incidence in women would correspond to physiological characteristics, particularly hormonal [37]. In addition, it has been demonstrated that this proportion has risen in recent decades, to the detriment of women [38]. This could be due to the recent increase in female smoking as well as the commercialization of birth control pills or to factors that could impact on epigenetic mechanisms that are currently unknown (Figure 2). To date, no environmental predisposing factor has been identified with certainty. On the other hand, in recent years there have been important advances in genetics.

## 5. MS a Multifactorial Disease

Several factors have been the subject of many studies and therefore benefit from more arguments regarding their role in the development of MS: vitamin D (associated with sunlight), viruses, and the genetic component of MS.

### 5.1. Sun and Vitamin D

As mentioned above, there is a heterogeneous global distribution of MS prevalence [14]. The most apparent correlation to latitude is sunlight. Several studies have shown an inverse correlation between MS risk and sun exposure before disease onset [8,9,10,11]. Subsequently, other studies have shown a highly significant inverse correlation between the prevalence of MetS and the annual rate of ultraviolet (UV) received by different regions [7,10]. This inversely correlated phenomenon can be biologically explained by the production of vitamin D, with exposure to light being necessary to produce this vitamin [39]. Some studies have highlighted the protective effect of vitamin D on the development of MS [12,13]. It is now accepted that vitamin D acts on the immune response, inhibiting the production of proinflammatory cytokines [14] and facilitating the development of regulatory T lymphocytes [12]. In addition, some studies in the mouse model of MS, experimental autoimmune encephalomyelitis (EAE), indicate that vitamin D may be beneficial in preventing and treating MS [14]. It is also interesting to note the presence of excess patient births during the summer, which could be related to pregnant women’s low vitamin D levels during winter [40].

### 5.2. Viral Exposure

Two viral hypotheses in MS have been proposed [13]:

- The hygiene hypothesis postulates that a succession of infections by different pathogens during childhood would protect against the disease. At the same time, first contact with these same viruses in adulthood would trigger MS. This hypothesis is currently the most unifying.
- The prevalence hypothesis postulates that a more common pathogen in regions with a high prevalence of the disease is the cause of the disease. This pathogen would be globally present and cause persistent asymptomatic infection until the onset of symptoms in rare cases several years after the primary infection.

Many viruses have been suspected of causing the disease, such as the measles virus, and certain herpes viruses, such as EBV, which are now the subject of numerous studies [13,14]. However, none have proven their real involvement.

### 5.3. The Genetic Component of MS

**Figure 3:**
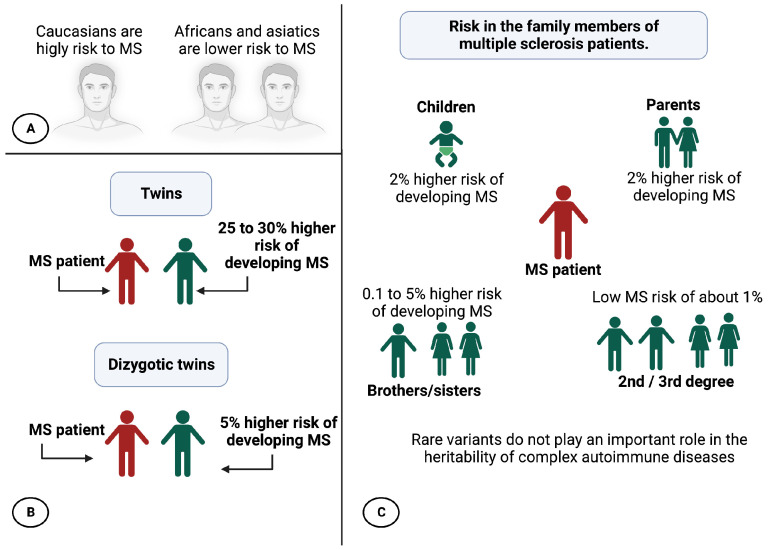
Genetic predisposition to MS. (**A**) Risk of MS by ethnic background; (**B**) Possible MS in twins; (**C**) MS risk in patients’ family members. Created with Biorender.com.

At the genetic level, epidemiological studies have shown a difference in MS predisposition according to ethnicity. Caucasians are highly susceptible to the disease, while MS is less common in Asian and African populations (Figure 3A) [6,14,41].

Family studies have shown that the risk of developing MS for a patient’s twin is increased by 25–30% compared to the general population. In the case of DZ twins (Figure 3B), this risk is only increased by approximately 5%. On the other hand, approximately 20% of patients have at least one affected relative. The risk of developing the disease for a first-degree relative of a patient increases from 0.1% to 5% for brothers/sisters, 2% for parents, and 2% for children. For 2nd-degree and 3rd-degree relatives, this risk is lower, close to 1% (Figure 3C), but it is still higher than that of the general population (between 0.1% and 0.2%) [16,34,36]. Therefore, all these arguments support the participation of genetic factors in the appearance of MS.

Two hypotheses have been proposed to explain the etiology of common diseases such as MS [41]:

- The common disease/common variants hypothesis [15,16]: the genetic predisposition to common diseases is determined by a few genetic variants, frequent in the population (frequency greater than 5%), each of which would confer only a low risk of developing the disease, with an odds ratio (OR) of between 1.1 and 1.5.
- The hypothesis of multiple rare variants or hypothesis of heterogeneity [17]: the genetic predisposition to frequent diseases is due to a combination of rare variants in the population (frequency between 0.1% and 5%) but each with a strong effect, with an OR of between 1.5 and 20.

Although these hypotheses initially seemed totally exclusive, a mixed model seems the most plausible theory today. Predisposition to common diseases would combine a common history determined by a few common variants and heterogeneity determined by several rare variants [18]. Each of these variants would not be necessary or sufficient to develop the disease, it would be the interaction between all these variants that would give rise to the genetic predisposition to the disease. According to these two hypotheses, simulation studies have made it possible to estimate the number of variants necessary to develop a common disease. Under the common disease/common variants hypothesis, 20 to 100 common risk variants would be needed. In contrast, under the multiple rare variants hypothesis, several hundred to several thousand rare variants would be needed to develop the disease [42].

The study by Hunt et al. casts doubt on the likely role of rare variants in complex diseases. Association analysis performed on the sequencing and genotyping data of the exons of 25 genes associated with at least two diseases with an autoimmune component (between MS, type I diabetes, Crohn’s disease, celiac disease, psoriasis, hypothyroidism), comparing a population of 25,000 patients and 17,000 controls, seems to indicate that rare variants do not play an important role in the heritability of complex autoimmune diseases, which would be due to the interaction of several frequent variants with weak effects [43].

## 6. The *Locus* of the MHC and MS

The association of MS with the genes of the MHC or the HLA region has been known since the early 1970s. The genes of this region are divided into three classes: HLA-class I, II, and III and encode highly polymorphic surface glycoproteins involved in the immune recognition of self and non-self [44]. They are found on antigen-presenting cells’ (APCs) surfaces and activate T lymphocytes (LTs). In the early 1970s, serologic analyses targeting MHC identified an association between MS and class I alleles A3 [45] and B7 [46]. The association of HLA-A3 has been shown to be secondary to that of HLA-B7, which, in turn, has been shown to be probably secondary to the association of the class II HLA-DR2 and DQw6 alleles with MS [5,47,48]. Today, the association of the class II haplotype: HLA-DQB1*0602, HLA-DQA1*0102, HLA-DRB1*1501 (corresponding to the serological alleles HLA-DR2, DQ6) is established [49]. Numerous studies have confirmed the association of the disease with the HLA-DRB1*1501 allele [50]. People who carry this allele have a three to four times higher risk of developing the disease, making it the main predisposing genetic factor for MS. However, a strong linkage disequilibrium within the HLA region makes it difficult to identify the genes predisposing to the HLA-DQB1*0602, HLA-DQA1*0102, and HLA-DRB1*1501 haplotype [51]. The HLA-DRB1*15 allele is subject to gene interaction (or epistasis) phenomena in MS. There is a risk associated with certain allele combinations of the HLA-DRB1 locus. The highest risk is attributed to HLA-DRB1*15 homozygotes, while the HLA-DRB1*14 allele has a protective role against the disease. Furthermore, the HLA-DRB1*08 allele only modestly increases the risk of developing MS, and while associated with HLA-DRB1*15, the risk of MS doubles [52,53]. The biological mechanism of the HLA-DRB1 gene in the development of MS is still not well understood. Studies of the protein structures of the HLA molecule seem to show that the highly polymorphic residues of HLA-DRB1 would act on the shape and charge of the antigen-binding site, and therefore could affect the efficiency of the presentation of these antigens to cells that are immune (Table 2) [52].

### 6.1. Linkage Studies

The first genetic analyses performed in multiple sclerosis were anonymous genome analyses based on the linkage analysis of the microsatellite markers in multiplexed families. Although they allow the identification of regions of interest outside the MHC, their participation in replication studies cannot be confirmed [41,54,55]. These studies have focused on designing genome-wide arrays aimed at identifying causal variants. However, the underlying causal variation at most of these associated loci has not yet been identified.

There has been progress in the discovery of genetic variations associated with the risk of MS. In 2019, the International Multiple Sclerosis Genetics Consortium conducted a study of genotype in peripheral immune cells and microglia, finding 233 statistically independent associations with MS susceptibility spanning the entire genome. Of these, 32 of these associations are located in the MHC, 1 on the X chromosome, and the remaining 200 associations were on the autosomal non-MHC genome. This same study estimates 48% of the heritability for MS [56].

To identify variants consequential in MS, the regions surrounding the associated variants must be shortened. The two main reasons that could explain these failures are the limited number of markers (microsatellites or SNPs) used in these studies and the limited number of families available for analysis. There are several strategies for this process, known as fine mapping, including heuristics based on link disequilibrium (LD) patterns, penalized regression, and a Bayesian methodology [57].

### 6.2. Association Studies

Candidate gene studies made it possible for the first time since 1972 to identify an HLA locus-independent MS predisposition gene: the IL7RA gene [58,59]. At the same time, an anonymous screening of the genome, analyzed by association and published by the IMSGC (International Multiple Sclerosis Genetics Consortium, i.e., the International Consortium on MS Genetics, which then brings together only American and English teams) allows the identification of two genes that predispose to MS: the IL7RA gene and the IL2RA gene [55]. These encode, respectively, an interleukin 7 receptor subunit and an interleukin 7’ receptor subunit, Interleukin 2. The GWAS [60,61,62] and a meta-analysis (called meta v1.0) [63] that combine several GWAS, allow the identification, with certainty, of 16 chromosomal regions that predispose to the disease, and another 10 that are potentially associated with MS. However, the GWAS has published a maximum of 1600 patients and 3400 controls and the largest meta-analysis included 2600 and 7200, respectively, representing only low statistical power. In 2011, the IMSGC performed the largest GWAS in MS. The consortium now brings together 23 research teams from 15 countries (Germany, England, Australia, Belgium, Denmark, Spain, USA, Finland, France, Ireland, Italy, Norway, New Zealand, Poland, and Sweden). The rationale for the study was published by Sawcer et al. in 2008 [64]. Based on these estimates, the required study sample size for the detection of a predisposing variant with a frequency of 10% and an OR of 1.2 is approximately 10,000 patients and 10,000 controls. Thanks to the pooling of the biological resources of the 23 teams, the IMSGC published in August 2011 results related to the analysis of 450,000 SNPs in 9772 patients and 17,376 controls [41]. This study increased the number of known non-HLA predisposing regions for MS to 52. The IMSGC project led:

- to the confirmation of the association of 23 of the 26 suspicious chromosomal regions, highlighted by the studies published between 2007 and 2010;
- to the identification of 29 new regions;
- to the highlighting of five potentials. Each of the identified variants confers only a low risk of developing the disease, 1.1 to 1.3 times higher than that of a non-carrier individual.

It is interesting to note that 21 of the 57 variants revealed by this screening are predisposing factors common to other so-called autoimmune diseases, such as type I diabetes, celiac disease, rheumatoid arthritis, ulcerative colitis, Crohn’s disease, or psoriasis. This finding reinforces the hypothesis of a common genetic architecture in these complex diseases. However, even if they are common to several dysimmune diseases, the directions of the associated effects are not always the same. Thus, a risk allele for one disease can be a protective allele for another disease [65]. Some studies have focused on the percentage of risk variants shared between different dysimmune diseases but did not consider the effects associated with these variants. Sirota et al. integrated into their study the directions of the effects of the variants associated with these diseases and hypothesized that certain loci would predispose to dysimmune diseases in general and that others, due to their protective or risk effect for one or diseases, would determine the specific predisposition of an individual to a disease (Table 3).

The GWAS represented a real advance in the knowledge of the genetic predisposition to multiple sclerosis. Today, 52 genetic markers of predisposition to the disease are known and unanimously recognized, as well as 5 potential ones, excluding HLA. However, the results obtained explain only 17% of the genetic part, although the project was designed to identify 80%. This is not unique to MS. Indeed, GWAS studies have been carried out for many years in different diseases and phenotypic traits and although all of them have been designed to identify a large part of the genetic part of the diseases, the results obtained (1.5% to 50%) do not allow them to be explained. Thus, the 32 loci identified in Crohn’s disease and the 18 loci identified in type 2 diabetes explain, respectively, 20% and 6% of the genetic share of these diseases [66].

### 6.3. Heritability Calculation

The calculation of the heritability of a risk or a predisposition is estimated from genetic correlations between siblings. The 61 variants identified (57 non-HLA and 4 HLA) by the IMSGC GWAS give first-degree relatives of a patient a relative risk of developing the disease of 1.58. In the Swedish population [67], the overall risk for siblings is 6.3. The 61 variants, therefore, explain approximately 25% (1.58/6.3 × 100) of the relative risk of a relative of developing MS.

The heritability calculation is based on the susceptibility threshold model where the total variation in the risk scale is 1 in the population. Under the normal distribution, considering the prevalence of the disease in the general population (K = 0.001), this model identifies patients as individuals with a greater predisposition than T = 3.09. Considering a relative risk for siblings of 1.58, conferred by the 61 variants, and assuming a null effect of environment, the heritability h is 38%. Each identified variant explains an individual relative risk (RRind) of developing the disease calculated according to its OR (OR), the frequency of the allele at risk (f) and heritability (h) calculated from the correlation between siblings, that is, 38% [41].

RRind = 0.5 × f(1−f) × OR2/h2

The global relative risk explained by all the identified variants is then the sum of the individual relative risks. Based on these calculations, it is estimated that the 4 HLA variants identified by the IMSGC GWAS explain 10.5% of the genetic part of the disease, while the 48 non-HLA variants explain 6.5%. The GWAS results explain 17% of the genetic component of multiple sclerosis. It is in this context, the notion of “missing heritability” [68]. This missing heritability thus corresponds to the genetic part that remains to be identified, since the risk variants, highlighted up to now and considered individually, do not make it possible to explain the entire heritability of the disease. As described by Maher [68], Manolio et al. [66], and Marian [69], and in addition to identifying the effects of individual variants, the lack of heritability could also be explained by:

- Unidentified gene interactions (also called epistasis phenomena);
- The importance of gene interactions.

### 6.4. Epistasis

It is the phenomenon by which the effect of one variant is affected by another. Unlike quantitative traits, mainly due to additive effects rather than epistatic (non-additive) effects, complex diseases are due to a combination of these two effects. Therefore, the interaction between the predisposition variants highlighted by GWAS could explain part of the lack of heritability of complex diseases. In MS, an epistasis analysis of HLA-DRB1 variants revealed combinations with varying degrees of MS risk. Thus, although the HLA-DRB1*15 allele is commonly considered the highest-risk allele for MS, with an allele risk of 3, its combination with the HLA-DRB1*14 allele decreases the risk associated with the single allele. On the other hand, the association of HLA-DRB1*15 with HLA-DRB1*08 confers a relative genotypic risk of 6.5, almost as high as the HLA-DRB1*15 homozygote [53].

### 6.5. Gene-Environment Interactions: GxE

A biological interaction is defined as the joint effect of two factors that act together in a physical or chemical reaction and the common participation of at least two factors in the same mechanism that leads to the development of the disease. Risk factors are said to be “interacting” if the effect of one risk factor depends on the other. Taking GxE interactions into account for the identification of genetic risk factors might increase the power to detect genes with weak individual effects, especially if the gene only has an effect in each subgroup of patients, defined by precise environmental exposure [70]. In the case of MS, a vitamin D level/HLA-DRB1 locus interaction associated with the disease has been identified. HLA-DRB1 is the only HLA locus capable of possessing a vitamin D response element (VDRE, vitamin D response element) in its promoter. Interestingly, all HLA-DRB1*15 haplotypes include the VDRE in the promoter. The HLA-DRB1 promoter is less conserved in the other haplotypes not associated with MS. Functional studies have shown that the presence of a VDRE in the HLA-DRB1 promoter influences the expression of this gene [53].

### 6.6. Lambda-S Parameter

**Figure 4:**
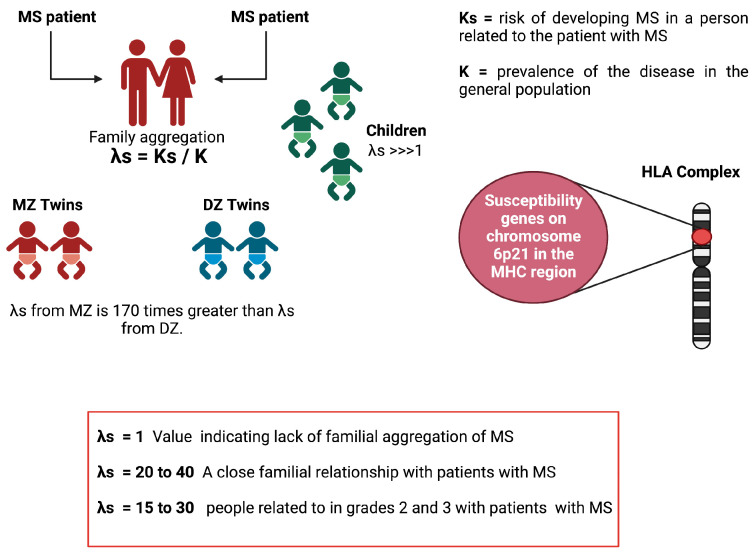
Family aggregation values in different family contexts. Increased risk of developing multiple sclerosis in MZ twins and first-degree family members of an MS patient. Created with Biorender.com.

A tool to measure this family aggregation is the λs. This parameter is defined as the relationship between the risk of developing MS in a person related to the patient with MS (Ks) and the prevalence of the disease in the general population (K = 0.1–0.2%): (λs = Ks/K) [71]. Thus, a value of s equal to 1 would indicate the absence of a familial aggregation of disease. In MS, this value of λs generally varies between 20 and 40 for close relatives of the person with MS. Using standard genetic epidemiology methodology and correcting for the age factor, it has been shown that people related in the 1st-degree, 2nd-degree, and 3rd-degree to a person with MS have a higher risk of developing the disease compared to the general population. The risk thus increases from 0.1% to 3% for a first-degree relative (5% for brothers and sisters, 2% for parents, and 2% for children), that is, a λs of the order of 15 to 30 for people related. In grades 2 and 3, this risk is lower (approximately 1%) but still higher than in the general population. However, these data are insufficient since they do not allow the distinguishment between the weight of genetics and that of the family environment [72]. In addition, work done with adopted children and half-brothers/sisters supports the concept that genetic factors are primarily responsible for the familial clustering of the disease [71]. Although adopted children have lived since childhood with a person with MS, they did not present a higher risk of developing MS than the general population (λs = 1) [73]. Studies were also carried out with half-siblings, which made it possible to verify the effect of sharing genetic inheritance on the risk of developing the disease (half-siblings share only 25% of their genetic information, while children who share the same parents have 50% of their genetic information in common). Half-siblings of a child with MS have been shown to have a significantly lower risk than “full” siblings (1.3% vs. 3.5%, *p* < 0.001) [74]. Studies in families where both parents have MS have shown that children of these couples are at significantly higher risk compared with children of families where only one parent has MS [75]. Twin studies have shown the critical role of genetics in the disease. While monozygotic (MZ) twins share 100% of their genetic information, dizygotic (DZ) twins share only 50%, such as “unique” siblings. The concordance observed in MZ twins is just as significantly higher than that observed in DZ twins or single siblings (concordance equivalent to 25% for MZ twins, 5% for DZ twins, and 3% for siblings) [76]. Thus, in MZ twins, the risk of recurrence is approximately 34%, conferring a 170-fold increased risk (λs) [72]. Furthermore, the importance of the gender of the twins was underlined, as the authors demonstrated that the observed risk difference between MZ twins and DZ twins was not found in male twins. However, despite having identical genetic information, most MZ twins are discordant for MS (approximately 75% are discordant), suggesting the importance of non-genetic factors in the etiology of the disease [76]. In addition to these epidemiological data, several susceptibility genes are known to date. Since the early 1970s, several susceptibility genes located on chromosome 6p21 in the MHC region have been described (Figure 4).

### 6.7. Epigenetic Phenomena

**Figure 5:**
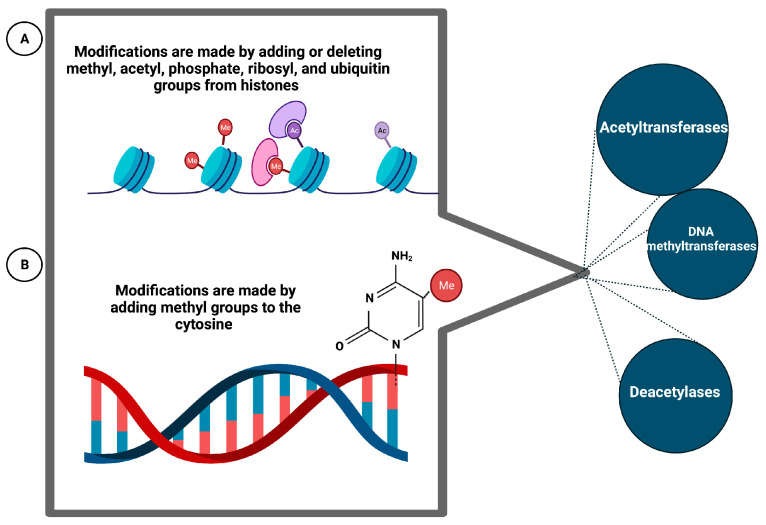
Biochemical mechanisms that alter DNA and histone linked to the development of MS. These changes are originated as a response to the environment or developmental cues. (**A**) This section shows the chemical changes that affect histones. (**B**) This section shows straight methylation on DNA. Created with Biorender.com.

Epigenetics consists of the regulation of transcription by transmissible and reversible modifications of DNA or histones without alteration of the DNA sequence. These modifications are made by adding or deleting methyl, acetyl, phosphate, ribosyl, and ubiquitin groups from histones or by adding methyl groups to the cytokines on the CpG islands of promoters. These additions/deletions occur via histone acetyltransferases/deacetylases or DNA methyltransferases (DNMTs, DNA methyltransferases). Epigenetic regulation is a dynamic process that can change and adapt in response to environmental or developmental cues. The influence of epigenetic factors in MS could be confirmed by the low concordance rate observed in MZ twins (26%). This suggests, among other things, that environmental factors or the effect of these factors on DNA transcription may play a role in MS. Thus, the inactivation of the X chromosome, a consequence of epigenetic mechanisms, could explain the unfavorable sex ratio for women observed in MS. Furthermore, in general, naïve T cells, when differentiated into TH1 and TH2 type cells, have been shown to exhibit DNA methylation. Low DNA methylation of T cells has been associated with the induction of autoreactive T cells. For example, T cells from rheumatoid arthritis patients have been shown to exhibit decreased DNA methylation due to reduced DNMT activity [77]. In MS, a study of the T cell epigenome of three pairs of MZ twins with discordant disease identified no significant differences in CpG island methylation (Figure 5) [78].

In recent years, more specific studies, restricted to genes whose functional effects are known in experimental models, have also made it possible to identify predisposing genes. This is how it has been shown that a haplotype of the VAV1 gene is associated with a predisposition to MS. VAV1 encodes a signal-transducing protein in lymphocytes and is in a genomic region known to regulate EAE in rats. Analysis of seven cohorts representing 12,735 individuals identified a two-SNP haplotype, located in the first intron of the VAV1 gene, associated with MS predisposition. The risk combination is associated with increased VAV1 expression in MS patients. VAV1 expression also correlates with TNF and IFN-gη expression in peripheral blood cells and cerebrospinal fluid [79]. These functional studies of candidate genes by function constitute a complementary approach to GWAS. As was the case for the IL7R and IL2R genes, they may allow the identification of new MS predisposing factors.

## 7. Genetic Changes in MS, Connections and Mechanism of Action

As previously stated, translating data such as GWAS into biological functions has been challenging because the deficiency is the generalized linkage disequilibrium (LD) throughout the human genome, which makes it challenging to identify the true causative variants. Some studies have employed various experimental systems to study the biological functions associated with MS risk variants. The available functional data point to a transcriptional hypothesis that risk variants increase the propensity to develop MS by primarily affecting the expression of associated genes.

The SNP rs6897932 found in exon 6 of the IL7R gene, which codes for the receptor’s transmembrane domain, was the first probable causative variant discovered in MS. The relative quantities of the protein’s soluble and membrane-bound isoforms are impacted by this SNP’s disruption of an exonic splicing silencer [58]. The rs2523506 SNP situated in the DDX39B5’UTR increases MS risk via lowering the translation of DDX39B mRNA, according to additional evidence that the RNA helicase DEAD box polypeptide 39B (DDX39B) is also a powerful activator of IL7R exon 6 [80]. The IL2RA gene intronic SNP rs2104286 has a similar effect to that mentioned. By encouraging the development of larger quantities of its soluble form, this risk variant does indeed change the IL2RA protein’s soluble/membrane-bound ratio [81]. The intronic SNP rs1800693 in the TNFRF1A gene is another well-studied instance. In this instance, the risk allele encourages exon 6 skipping with the development of a new soluble version of the tumor necrosis factor (TNF) receptor that may limit TNF signaling within cells, somewhat resembling the effects exacerbating TNF-blocking medications during MS [82]. The EVI5 protein’s coiled-coil domain saw alterations in the surface hydrophobicity due to the non-synonymous exonic SNP rs11808092 at the ecotropic viral integration site 5 (EVI5), which, in turn, affected the EVI5 interactome. A key finding of the study was that the risk allele-carrying EVI5 protein interacts with sphingosine 1-phosphate lyase (SGPL1), a key enzyme in the formation of the S1P gradient that is important for the adaptive immune response and the treatment of MS [83].

The identification of cellular pathways dysregulated by disease has been significantly helped by recent developments in bioinformatics and computer-based analysis techniques.

Additionally, the computation of the input genes overrepresentation in particular gene ontologies (GO). GO analysis of risk genes points to their involvement in pathways such as KEGG, the JAK-STAT signaling pathway, acute myeloid leukemia, and T-cell receptor signaling, as well as macromolecule metabolic processes [84]. They also discovered that the NF-kB cascade was highly related to MS risk genes by expanding the pathway analysis to the 110 non-MHC variations discovered after immunotyping [85,86].

Specific genetic variations that increase MS susceptibility also impact the disease’s progression and clinical appearance. Most genotype-phenotype investigations concentrate on the HLA alleles because they are the earliest genetic predictor of MS risk and exert the most significant influence on MS susceptibility. Masterman et al. state that HLA-DRB1*15:01 carrier status is consistently linked to a younger age at the disease start [87]. Additionally, glatiramer acetate, an immunomodulatory medication whose mechanism of action involves binding to MHC class II molecules as a first step is affected by HLA-DRB1*15:01 [88]. Additionally, as measured by magnetic resonance imaging (MRI), this genotype accelerates the development of MS brain pathology regarding reduced brain magnetization transfer and T2 lesion load [89].

According to Healy et al. the protective allele HLA-B*44:02 preserves brain volume and lessens the impact of T2 hyperintense lesions [90]. A recent study examined the HLA locus’s global impact on clinical and MRI results. They computed the cumulative HLA gene load (HLAGB), the effect of having various alleles in various HLA genes, in 455 European-descent controls and 652 MS patients with comprehensive phenotypic data. Previous research revealed that in women with RR-MS, higher HLAGB scores are linked to an earlier age of onset and atrophy of the subcortical gray matter component. For subcortical gray matter atrophy, HLA-B*44:02 showed a nominally protective effect [91].

To understand the genetics of these immunologically complex diseases, such as MS and NMOSD, the aggregate genetic risk score, and its ability to integrate genetic contributions, you must compare NMOSD patients and MS patients and, thus, analyze genetic heterogeneity.

## 8. Genetic Factors of the NMOSD

The NMO spectrum disorder comprises a variety of disorders characteristic of an acute inflammatory response in the optic nerve and spinal cord. NMOSD is a complex and multifactorial disease. Most cases of this disorder are sporadic and approximately 3% are familial; these have similarities in terms of clinical manifestation, age of disease onset, and effects based on gender. On the other hand, most patients with NMOSD are positive for the aquaporin-4 (AQP4+) antibody, others for the myelin oligodendrocyte glycoprotein antibody (MOG+) and the rest are not, the so-called double seronegative [92]. It is unclear whether these different serotypes of NMOSD patients belong to the same disease continuum or are other diseases with the same clinical expression [92,93]. There have been records of NMOSD cases in identical twins in early adulthood and NMOSD manifestations in two siblings at three years of age, with a shared HLA haplotype [93,94]. More recent studies describe familial cases of NMOSD in parent–child, sibling, and aunt–niece pairs, with a predominance of women (80%). Several reported cases have had maternal or paternal transmission, and >75% of cases had AQP4-IgG. The observation of familial transmission in NMO suggests a complex genetic etiology for this disorder [93]. A descriptive and retrospective analysis of the reported cases of NMOSD in a population from Western Mexico (Jalisco, Mexico), where 55.2% of the patients were AQP4–IgG + and 14.9% were AQO4-IgG -, an adjusted prevalence was found of 0.71/100,000 and an adjusted incidence of 1.87/1,000,000 person-years. Most of the patients were female (74.6%), so the authors infer that the incidence is higher than in Caucasian populations [26]. NMOSD has a high prevalence in non-European populations, where ethnicity influences the frequency of NMOSD in Latin America. Because the contemporary mestizo Mexican population resulted mainly from miscegenation where Native American ancestry stands out, this could tell us about an increased risk of developing NMO in the Mexican population (Table 4) [95].

Recent studies have reported associations between NMOSD and genetic variations in the HLA region of the genome on chromosome 6, especially in class II alleles, which show ethnic and geographic differences. The DRB103:01 allele has been associated with NMOSD in European, Brazilian, Afro-Caribbean, and Mexican patients; DRB116:02 in southern Han Chinese, Japanese, and southern Brazilian patients; DQB104:02 in a cohort of European descent, and DRB104:05 in southern Brazilians [94,95]. Candidate gene studies have reported associations with variations in non-HLA genes, such as AQP4 and others involved in immune function (PD-1, IL-17, IL-7R, CD6, and CD58) [92,98]. Of these, only AQP4 gene variation in various populations has been analyzed by the sequencing of promoter and/or coding regions of the gene to identify variants involved in NMOSD pathogenesis. However, the association of AQP4 gene variation with NMOSD remains uncertain, with inconsistent findings across populations [95]. Estrada et al. [96] found two independent SNPs (rs1150757 and rs28383224 in the MHC region) associated with AQP4–IgG+ positive NMOSD. However, patients’ susceptibility genes and related pathways have not yet been identified with NMOSD negative for AQP4–IgG+, and in those that are positive, there is another genetic variant of a single nucleotide located in HLA-DQA associated with NMO AQP4–IgG+ [94].

Genetic variants in the TNXB gene encode for the members of the tenascin family, which are extracellular matrix glycoproteins with anti-adhesive effects. This protein functions in matrix maturation during wound healing, and its deficiency has been associated with connective tissue disorders. This gene is in the class III MHC region on chromosome 6 (TNXB Tenascin XB [Homo sapiens (Human)]—Gene—NCBI, n.d.). Whole genome sequencing studies have revealed that carriers of the risk allele (A) for the rs1150757 single nucleotide genetic variant in the TNXB gene have an increased risk of developing NMO. This is a synonymous variant in the TNXB gene, and it is located 26 kb from the C4A and C4 genes. It can also increase the risk of presenting NMO up to 4.66 times [96]. In the Mexican population of Los Angeles, it has been identified that 2% of the subjects are carriers of the minor allele A [95].

The rs28383224 variant, located in the HLA-DQA gene, an important member of the class II MHC family found on chromosome 6p21, could be a potential prognostic biomarker for NMOSD. Abnormal HLA-II expression can result in an insufficient immune response or an autoimmune reaction, leading to various diseases, including NMOSD. This single nucleotide genetic variant has a strong association with susceptibility to NMOSSD in the European population (OR = 2.6) [94]. On the other hand, Estrada et al. [96] demonstrated that HLA-DQA1 (rs28383224) was shared between AQP4-positive and AQP4-negative NMO, suggesting that there is at least one common genetic determinant for both groups. However, Li et al. [97] found no shared genes between the AQP4–IgG+ positive and negative group. Due to the complexity of the MHC region, larger studies will be needed to determine the role of HLA alleles and to understand the effect of these haplotypes on the NMOSD subgroup. In a study conducted in the Han ethnic Chinese population, Zhou et al. [94] found no statistically significant evidence of the association with the single nucleotide genetic variant rs28383224 present in the HLA-DQA1 gene in subjects with NMO. However, in the Mexican population residing in Los Angeles, California, 36% of people carry the A allele of the single nucleotide genetic variant rs28383224, so it will be of great importance to study the prevalence of the risk allele in the Mexican population [95]. There are also genetic variants of a single nucleotide present in the AQP4 gene. This gene is responsible for encoding a member of the aquaporin family, which functions as selective water channels in the plasmatic membranes of multiple cells. In this case, it is predominant in the brain and participates in water homeostasis. Subjects carrying the T allele of the single nucleotide intronic variant rs2075575 in the promoter region of the AQP4 gene cause downregulation of the AQP4 gene. This has been observed more frequently in subjects with NMO and increases up to 2.89 times the risk for its pathophysiological development for APQ4-Ig NMO in the Japanese population [92]. The presence of the A risk allele for the rs2075575 variant has a frequency of 40% in the Mexican population residing in Los Angeles, California. There are associated genetic variants present in genes that regulate the immune system and water channels in the brain, and some of these have an important presence in the Mexican population. It is necessary to elucidate the pathophysiological role played by each of them and their prevalence in our mixed-race and Native American population to determine the genetic risk that individuals could have in the presence of environmental risk factors.

Different studies show that familial clustering and the high prevalence observed in Asian populations indicate a possible genetic influence on the risk of NMOSD. A CNPY3 variant was found by performing whole exome sequencing (WES) for different families with NMOSD [99]. Two patients (II-2, III-2) from one of the families were identified as carriers of the CNPY3 mutation (c.155 G > A; p. C52Y) [99]. Subsequently, in this same population, a new familial NMOSD variant was identified and showed the C52Y mutation in CNPY3 downregulates the subcellular localization of a series of TLRs [98].

At the end of the day, we can ask ourselves: How much do we know about neurodegenerative demyelinating inflammatory diseases of the nervous system? It is a question for our generation and the ones that follow.

## 9. Conclusions

MS and NMOSD are diseases that share similarities due to direct affectation by the immune system. Thanks to gene-wide association studies, it is possible to identify the relevance of the genetic structure of proteins associated with some biological pathways, which seem to predispose to be affected by any of the diseases. These GWAS studies have made possible the comparison of the characteristics of certain protein gene expression polymorphisms where they have been assigned a statistical cutoff to be considered predisposing to the disease, such as the PRKCE, BCL2, and TYK2 genes, in the case of MS. It is important to recognize that the changes within the polymorphisms of these genes are not synonymous with suffering from the disease, as in monogenic diseases. Rather, it has been allowed through genome databases to compare certain SNPs that have been identified to be more frequently expressed in sick patients. Epistasis and environmental factors could be the cause of MS or NMOSD, that is, the interaction of the proteins generated with these polymorphisms can interact with the cellular environment or even with other proteins of the same pathway and could be the cause of the disease. These theories have been proposed due to the level of complexity and association found in healthy controls, which, despite expressing the protein with polymorphism, did not develop the disease. NMOSD-associated polymorphisms have been localized to the HLA region of the genome on chromosome 6, especially in class II alleles, and other candidate gene studies have reported associations with variations in non-HLA genes, such as AQP4 and others involved in immune function (PD-1, IL-17, IL-7R, CD6, and CD58). This allows us to recognize the theory that certain clinicopathological manifestations of the same disease can be caused by dysfunctions in different points of assembly of the immune response and that the misexpression of some host antigens that are known as foreign by the immune cells can cause the development of the disease.

## Figures and Tables

**Table 1:** Summarized associated factors in MS pathogenesis.

Associated Factors of Multiple Sclerosis		
Environmental factors	Sunlight [4,6,7,8,9,10,11]	Inverse correlation between the prevalence of metabolic syndrome (MetS) and the annual rate of ultraviolet skin cancers related to exposure to sunlight were significantly less common in patients with MS than in matched controls, implying that greater exposure was protective against MS.
	Vitamin D [4,6,12,13]	- It has a protective effect on MS development. - Inhibits production of proinflammatory cytokines. - Facilitates development of regulatory T lymphocytes.
	Hygiene hypothesis [4,13]	The succession of infections by different pathogens during childhood would protect against the disease while a first contact with these same viruses in adulthood would trigger MS.
	Other factors [6,12,14]	Tobacco, fats (diet), chemical compounds
	Infective agents [15,16]	*Epstein–Barr, Acinetobacter* and *Pseudomonas* were significantly elevated in MS.
Genetic factors	The common variants hypothesis [15,16]	The genetic predisposition is determined by few genetic variants, frequent in the population (greater than 5%) but each confers a low risk of developing the disease (OR 1.1 to 1.5).
	The heterogeneity hypothesis [17,18]	The genetic predisposition is due to a combination of very rare variants in population (between 0.1% and 5%) but each confers a strong effect (OR 1.5 to 20).

**Table 2:** Alleles associated to Multiple Sclerosis.

Major Histocompatibility Complex Locus Associated with MS		
HLA-class I	Allele A3 Allele B7	A3 allele has shown to be secondary to allele B7. It has been probable secondary association with HLA-DR2 and DQw6.
HLA-class II	HLA-DR2 DQw6	HLA-DQB1*0602 is present in most populations with MS, it has not been possible to discern has any independent role in MS. HLA-DQA1*0102 and HLA-DRB1*1501 are the known association with the HLA class II DR2 haplotype.
	HLA-DRB1	HLA-DRB1 would act on the shape and charge of the antigen-binding site, and therefore could affect the efficiency of presentation of these antigens to cells. HLA-DRB1*15: The highest risk is attributed to HLA-DRB1*15 homozygotes, three to four times higher risk of developing MS. HLA-DRB1*08 allele only modestly increases the risk of developing MS.
HLA-class III	NOTCH4	A Japanese population genotyped for 3534 SNPs in the MHC region showed independent associations to both an HLA class III marker in the NOTCH4 gene.

HLA: Human leukocyte antigen, SNPs: Single-nucleotide polymorphisms.

**Table 3:** Predisposition genes HLA locus independent.

Association Studies		
Biological Pathway	Encodes	Characteristics
IL7RA	Interleukin 7 receptor subunit and 73 genes with putative relations.	Genes that had significantly associated single-nucleotide polymorphisms in an independent case-control dataset. IL7 SOCS *PRKCE* *BCL2* *TYK2* There are 7865 SPNs around those genes.
IL2RA	Interleukin 2RA	Encoding the alpha chain of the interleukin-2 receptor. It is not a specific marker of regulatory T cells. The effect of IL2RA might be better described by several SNPs rather than by a single one.

PRKCE: Protein kinase C epsilon, BCL2: B-cell lymphoma 2, Tyk2: tyrosine kinase 2.

**Table 4:** Genetic factor associated with NMOSD.

Genetic Factors NMOSD	
Ethnicity HLA [93,94]	DRB116:02 in southern Han Chinese, Japanese, and southern Brazilian patients. DQB104:02 in a cohort of European descent. DRB104:05 in southern Brazilians.
Non-HLA [20,92,93]	PD-1, IL-17, IL-7R, CD6, and CD58.
Familial cases [96,97]	Including siblings, parent–child, and aunt–niece pairs, with more than 80% of them being female. HLA-A*31, B*61, *51, DRB1*0802, and DPB1*0501. More than 75% of cases had AQP4-IgG.
Genomic studies [93,96,97]	HLA-DQB1*05:02-DRB1*15:01” haplotype has been higher in the NMO group compared with controls. The SNP rs1964995 in the MHC region as a risk locus. Additionally, genotyped eight SNPs in AQP4 in a group of AQP4-IgG-positive.

## Data Availability

Data are available on request from the authors.
